# The Royal Medical Benevolent College

**Published:** 1895-05-11

**Authors:** 


### The Hospital

An Institutional Journal of

The Medical Sciences and Hospital Administration, with

Vol. XVIII.—No. 450.] AN EXTRA NURSING SUPPLEMENT. [May 11, 1895.

### The Royal Medical Benevolent College.

A festival dinner will be given at the Imperial Institute on May 15th, which in the minds of all members of the medical profession should stand apart from, and occupy a higher position than, all the other festivals of the year, for its object is to aid a great medical charity which is founded for the purpose of helping the aged and the unfortunate among its own ranks, assisting their widows, and educating their orphans.

Mr. Balfour, who will occupy the chair at this festival, is a celebrated orator, and there can be no doubt that a great meeting will foregather on that occasion to hear him speak. We hope, however, that those whose good fortune it may be to join the feast will not be content to eat the dinner and enjoy the treat, but will consider that the eyes of the profession are fixed upon them, and that the interest of their brethren on this occasion will lie not in the words of Mr. Balfour, but in seeing how much money the dinner has produced for the benefit and extension of the charity in whose name it takes place. The duty of giving does not, however, solely attach to those who dine. Every member of the great profession of medicine should feel bound if not himself to give, at least himself to collect for this charity, which helps his less fortunate brethren, and may, perchance—for who knows the future—be the means of saving himself from penury and securing to himself some comfort in old age.

For it is plain to all that the struggle of life is increasing. It is no longer so easy as it was to retain a grasp of a steady-going practice; not only is the public more exacting, not only is more demanded for smaller fees, but there is a growing impatience with the old-fashioned, an urgent demand for fresh ideas and new devices, and thus it is becoming increasingly difficult every year for old men and for feeble men to retain their footing in the ranks of a constantly striving and constantly advancing profession. Never was a time when it was so important for those who still stand to hold out a helping hand to those who are down, and seldom indeed is it possible to find a means of rendering this help so usefully as by adding to the funds at the disposal of the Royal Medical Benevolent College, for the demands upon the charity have increased fourfold during the last eight years. This institution provides pensions for aged medical men, and the widows of medical men in reduced circumstances. In the buildings at Epsom an asylum has been provided for these old gentlemen, but by an Act of Parliament obtained last year the governing body has gained the right of giving augmented pensions without residence, and thus their powers of doing good are much enlarged. They only ask for means by which to do it.

Besides the pensions for the old the Royal Medical Benevolent College educates the young. The school generally known as "Epsom College" accommodates, in addition to fifty foundation scholars, about one hundred and eighty five resident pupils, who, if the sons of medical men, receive certain advantages in regard to fees. The school is well staffed and possesses every appliance for giving a first-class education. It is also well endowed with scholarships, among which may be mentioned ten at the hospital schools in London. So great, in fact, has been the success of the school that it is now found necessary to build a junior or lower school for 100 boys. It is to this education and to this opportunity of retrieving again the father's lost position, that the necessitous orphans of such medical men as would have been professionally qualified for pensionerships are admitted by the charitable operation of the Royal Medical Benevolent College. Fifty foundation scholars, in addition to the ordinary paying pupils, receive an education of the highest class, and are boarded, clothed, and maintained at the expense of the College, standing all the time on exactly the same footing as their *confrères*.

This truly is a charity of which the medical profession may be proud; but it is one that the profession should support with far greater liberality even than it shows at present.

The "Medical Register for 1894" contains the names of 32,637 medical practitioners. The annual report of Epsom for 1894 gives the total of subscriptions and donations as together amounting to £3,533. A little under two shillings apiece, and at least a third of this is given by laymen.

This should not be. We do not say that everyone should subscribe guineas, although those who can do should—and many of them. But there are other ways. In this matter we should take a leaf out of the book of our clerical brethren. Every medical man comes across people who, however chary they may be in paying bills, are willing enough to give. The annual reports and subscription lists which cumber the tables of many of our patients show how great is the stream of charity on the brink of which we stand. It only wants diverting. No one can believe that these good people are specially interested in all the missions, ragged schools, and soup kitchens to which they subscribe so freely; but they have been properly asked, and so they give. Why should they not be properly asked to give to the Royal Medical Benevolent College, to help to educate the young, who without such help will drop out of the position so hardly fought for by their fathers, or to help to keep from penury and distress those old people who have lived as gentle folk in their midst and as gentle folk have passed their lives among them doing good. Every one of the thirty thousand members of the profession should make himself a collecting agent for this important charity, and it will be strange indeed if, with so good a cause and such an agency, the Royal Medical Benevolent College does not quickly rise to increased prosperity and usefulness.

